# Comparing time focus with time importance for measuring future time perspectives in the context of pro-environmental values and outcomes

**DOI:** 10.3389/fpsyg.2023.945487

**Published:** 2023-02-27

**Authors:** Svein Ottar Olsen, Ho Huy Tuu, Ana Alina Tudoran

**Affiliations:** ^1^School of Business and Economics, UiT The Arctic University of Norway, Tromsø, Norway; ^2^Economics Faculty, Nha Trang University, Nha Trang, South Central Coast, Vietnam; ^3^Department of Economics and Business Economics, Aarhus University, Aarhus, Central Denmark Region, Denmark

**Keywords:** future time perspectives, pro-environmental behavior, willingness to pay, time importance, time focus, nomological validity

## Abstract

**Introduction:**

In the area of environmental psychology, time and the temporal perspective have often been used as an overarching framework to explain or predict environmental outcomes. This study aims to introduce a new Time Importance Scale (TIS) based on the attitude strength theory and to assess its nomological validity in comparison with the more established Temporal Focus Scale (TFS) in the context of consumers’ willingness to pay more for environmentally friendly products. The new TIS scale is short and simple to manage.

**Method:**

The study proposes competing plausible structural models testing alternative relationships between future time perspective (FTP) and environmental values using a nationwide representative survey sample of 633 Norwegians.

**Results:**

The results confirm the convergent and discriminant validity of the new TIS scale. However, the direct association between the TIS scale and willingness to pay for environmentally friendly products is weak or non-significant depending on the alternative models used to validate the nomological validity.

**Discussion:**

The new TIS scale provides evidence of a possible causal chain, FTP → environmental values → willingness to pay, with strong associations between the present TIS and hedonic values and between the future TIS and biospheric values. Environmental values are activated by FTP. In most cases, the new TIS outperforms the established TFS in nomological validity. Future research should validate our findings in experimental trials to demonstrate more substantial causal relationships.

## Introduction

The relationship between individuals’ time perspective (TP) and their pro-environmental behavior is highly relevant to researchers and practitioners when designing strategies to raise the public understanding of and concern about issues such as climate change (Milfont et al., 2012; Baird et al., 2021). Significant research nowadays focuses in particular on individuals’ attention to the future dimension of the time perspective (FTP: Kooij et al., 2018) and the temporal conflict between the present and the future, that is, immediate versus long-term/future consequences (Joireman et al., 2001; Milfont et al., 2012; Khachatryan et al., 2013). The empirical findings are mixed, depending on the theoretical approaches, the operational and methodological concerns, and the TP’s ability to explain/predict various types of environmental behavior (Bruderer Enzler, 2015; Baird et al., 2021; Shipp and Jansen, 2021). In their reviews, Kooij et al. (2018) and Baird et al. (2021) highlighted the lack of an integrative approach providing a more in-depth understanding of the FTP, the nature of the relationships among antecedents (e.g., individual differences), and the consequences of the FTP across specific behavioral domains as well as the extent to which the TP provides unique knowledge in the prediction of individual motivation, engagement, and behavior.

Joireman and King (2016) review contained similar ideas and suggestions for future research on individual differences in the FTP. They specifically called for more research on whether and how FTP constructs are theoretically and empirically related to evaluate the nomological validity of TP scales. In an editorial, Levasseur et al. (2020) recommended that scholars should specify their theoretical approach and explicitly position their conceptual choice in TP studies within the broader theoretical and empirical literature. Both Levasseur et al. (2020) and Mohammed and Marhefka (2020) requested more conceptual and methodological precision and consensus when different time perspectives are used in empirical studies.

This study addresses some of these critical issues and makes three essential contributions to the growing literature on the time perspective in pro-environmental behavior, with an emphasis on the FTP. Firstly, after discussing the theoretical perspective within the established time perspective frameworks, we define, test, and validate a new scale to assess the time perspective. The Time Importance Scale (TIS) is cognitive and is based on theories about attitude strength and importance (Eaton and Visser, 2008; Howe and Krosnick, 2017). The new TIS and the established Temporal Focus Scale (TFS: Shipp et al., 2009) share the same theoretical cognitive foundation. Both are short and simple to manage. Thus, the new TIS scale is validated against the TFS. The TFS is considered the most psychometrically sound of the most regularly used scales for assessing the time perspective (Mohammed and Marhefka, 2020). This study uses (personal) importance as the main attitudinal attribute to describe the focus or salience of the present and the future time perspective. Attitude importance is typically assessed through direct self-report questions about individuals’ subjective sense of concern, caring, and the significance that they attach to an (attitude) object or issue (Boninger et al., 1995, p. 160; Howe and Krosnick, 2017). However, TIS and TFS differ as cognitive constructs. Shipp et al. (2009) conceptualization of temporal focus (TFS) involves the frequency of thinking about time (“how often”) without specifying a particularly positive or negative evaluation (attitude) of individuals’ preferences or interests toward different time periods as in the TIS scale.

Secondly, we build on previous research by testing whether future time perspectives are related to one key pro-environmental outcome: the willingness to pay (WTP) for environmentally friendly products and services (Kooij et al., 2018; Qian et al., 2021). Thirdly, we enhance the idea of more integrative models examine the theoretical basis for the time focus and time importance approaches by evaluating their relationships buy using two essential but conflicting environment-relevant values, biospheric and hedonic/hedonistic values (de Groot and Steg, 2008), within a nomological structure. The theoretical debate centers on whether the FTP—as a cognitive and attitudinal construct—is activated by (environmental) values (Boninger et al., 1995; Fishbein and Ajzen, 2010; Murphy et al., 2020; Thelken and de Jong, 2020) or whether the FTP—as a more stable individual trait or tendency (e.g., personality)—activates environmental values and environmental behavior (Khachatryan et al., 2013; Joireman and Liu, 2014; Kairys and Liniauskaite, 2015). Based on two theoretical approaches we propose competing plausible structural models based on the two theoretical approaches. Overall, our study contributes to extend the use of the TFS in the context of environmental values and outcomes as well as in the social dilemma between hedonic and biospheric values (Balunde et al., 2019; Thelken and de Jong, 2020). The empirical test of the models and the nomological validity (Bagozzi and Yi, 1988) of the new scale are based on structural equation models using a nationwide representative sample of 633 Norwegians.

## Theoretical background and framework

Within social psychology, the terms time *perspective*, temporal/time *orientation*, temporal/time *focus*, and time *attitude* have often been used interchangeably within the overarching framework of the time perspective or temporal perspective (TP; Shipp et al., 2009; Mohammed and Marhefka, 2020). The definitions are based on theoretical constructs from trait-stable individual differences to less stable and more context/object-dependent motivational, cognitive, or attitudinal-based theories. Most definitions include the individuals’ associations with one, two, or three combinations of the different time frames: preferred (cognitive–motivational) orientation toward the past, present, or future.

The several theoretical approaches to the time perspective can affect the nomological network of the construct (antecedents and consequences), the time frame/horizons (e.g., past, present, and future), and the theoretical core of the construct. One of the most influential TP umbrella works is the Zimbardo Time Perspective Inventory (ZTPI: Zimbardo and Boyd, 1999). Based on Strathman et al. (1994) consideration of future consequences (CFC) work on individuals’ priorities for or consideration about potential distant outcomes of their current behavior and the extent to which they are influenced by these potential outcomes, the FTP has grown in popularity (Kooij et al., 2018). Apart from the ZTPI, the CFI is the most popular scale for measuring the (future) time perspective, especially in the area of health and environmental behavior (Kooij et al., 2018; Mohammed and Marhefka, 2020).

According to Shipp et al. (2009), individuals can have multiple temporal foci, they shift and control their foci, and they allocate their attention to various targets, periods, and situations. They suggest that individuals’ time focus is flexible, changeable (a state: within-person fluctuation), motivational, and cognitively based (Shipp, 2020; Shipp and Aeon, 2019). Their Temporal Focus Scale (TFS) differs from theories suggesting that individuals are predisposed to one time perspective in accordance with their personality traits and/or include different theoretical approaches in their conceptualization and assessment (e.g., ZTPI: Zimbardo and Boyd, 1999). However, the TFS is mostly used and validated within management, organizational research, individual differences, and positive psychology studies (Levasseur et al., 2020; Mohammed and Marhefka, 2020; Diotaiuti et al., 2021; Shipp and Jansen, 2021).

In the present research we include the attitude strength theory (Petty and Krosnick, 1995) within the established cognitive attention-based temporal framework and suggest a new Time Importance Scale (TIS) in the context of environmental behavior. Because our context is the area of willingness to pay more for pro-environmental products, our time frame is focused on the dilemma between the present and the future time perspective (FTP: Kooij et al., 2018). In line with Shipp et al. (2009), our research aims to evaluate a core facet of the time perspective that is theoretically robust, brief, and simple to use/measure in practical research settings.

### Defining time importance from an attitudinal strength perspective

Researchers have defined “attitude” as a general or overall, relatively enduring evaluation (judgement) of a stimulus object (Eagly and Chaiken, 1993). Attitudes are enduring and relatively stable in that they are presumed to be represented in long-term memory and are associated with specific objects, such as things, persons, oneself, issues, or concepts. The content of the attitude construct can be the cognitive beliefs, thoughts, and attributes that individuals associate with an attitude object and the feelings about or emotional associations with that object (Fishbein and Ajzen, 2010). In our study, we consider that (past and) present and future time horizons are attitudinal objects that individuals can evaluate differently depending on their individual time preferences, personalities, values, goals, emotions, experiences, or situations.

Our attitudinal perspective for studying the TP follows some of the previous attitudinal approaches to it, such as Nuttin (1984) Time Attitude Scale and the Adolescent Attitude Time Inventory developed by Worrell and his colleagues (AATI: Worrell et al., 2013). These approaches focus primarily on the direction (valence) and extremity components of the attitudes using statements such as “I am pleased with the present” and “I am not satisfied with my past,” for instance. The scholars also used other dimensions of similar to time orientation perspectives, such as “how often” the individuals think about time (frequency), how they perceive the relations between periods (time relations), and their attention to or focus on the past, present, and future (see Worrell et al., 2021 for a recent update). Unlike the time attitude perspective, Shipp et al. (2009) conceptualization of temporal focus (TF) involves thinking about time without specifying a particular positive or negative evaluation of the individuals’ preferences for or attitudes toward different time periods.

Personal or *attitude importance* is defined as the individuals’ subjective sense of concern, caring, and significance that they attach to an (attitude) object or issue (Boninger et al., 1995, p. 160; Howe and Krosnick, 2017). Importance is closely associated with other constructs, such as personal and self-relevance, vested interests, attitude or issue importance, personal or issue involvement, or simply importance (Boninger et al., 1995; Thomsen et al., 1995). However, the literature has distinguished between these facets of strength-related constructs (Eaton and Visser, 2008). Because various terms have often been used to describe the same or very similar phenomena, the present study uses (personal) importance as the main attitudinal attribute to describe the focus or salience of the present and the future time perspective (FTP). Given its status as a subjective cognitive evaluation, attitude importance is typically assessed through direct self-report questions about how personally important the attitude object is to the respondents, how deeply they care about the attitude object, and how concerned they are about the attitude object (Boninger et al., 1995). One advantage of using attitude strength (e.g., importance) is that it is useful in predicting and explaining specific behavioral outcomes (Howe and Krosnick, 2017). When an attitude is perceived as personally important, individuals use that attitude in the deliberative processing of information, making decisions, and performing the behavior (Boninger et al., 1995).

Consequently, this study contributes to the existing literature by introducing and defining *time importance* as the subjective sense of concern, caring, and significance that individuals attach to different time periods. As part of the attitude family, time importance has a positive and a negative valence and differs in extremity and strength. Individuals’ evaluation of time importance is considered to influence their thoughts, emotions, intentions, and behavior (Eaton and Visser, 2008; Howe and Krosnick, 2017). As a cognitive and knowledge-based attitude, time importance is dependent on specific situations, actions, and context (Fishbein and Ajzen, 2010) such as different pro-environmental behavior outcomes.

### Future time perspective and pro-environmental behavior

The different theoretical approaches for explaining and understanding the associations between time perspectives and behavioral outcomes vary somewhat depending on the behavioral context studied (Kooij et al., 2018; Murphy et al., 2020; Baird et al., 2021; Shipp and Jansen, 2021). For decades, the social dilemma approach has been a leading perspective in studies of the relationship between the future time perspective and environmental or sustainable behavior (Joireman et al., 2001; Milfont et al., 2012; Joireman and King, 2016). This approach describes the future time perspective as the interpersonal struggle between immediate (living for today) versus future long-term interests (temporal conflict) and/or the social conflict between individual self-interests versus collective social interests (see Olsen and Tuu, 2021 for a recent update). Thus, the individuals’ capacity and interest in thinking long-term may influence their decisions and choices when it comes to the purchase and use of environmental-friendly products and services.

When the ZTPI and CFC have been used to explain or predict pro-environmental attitudes, intentions, or behavioral tendencies, the findings have been equivocal (Khachatryan et al., 2013). For example, Bruderer Enzler (2015) found non-existent, low, and mixed relationships between CFC-immediate/future and 17 different environmentally friendly behaviors (turning off lights, regulating room temperatures, using a solar panel or heat pumps, etc.). In the literature, the predictive validity of the attitude-based time perspective approach (i.e., temporal focus) has received less attention (Kooij et al., 2018; Mohammed and Marhefka, 2020). General time focus, like the time attitude approach, has a non-significant, varying, or low predictive ability for more specific behaviors, such as alcohol-related outcomes, according to the limited research conducted (McKay et al., 2018, 2021). Furthermore, broader definitions of the TP (e.g., ZTPI and CFC) or assessments of the TP framed within a specific behavioral domain (e.g., health/food behavior) predict or explain variations in behavioral outcomes more precisely (Murphy et al., 2020; Pozolotina and Olsen, 2020). The results of existing studies are inconsistent and inconclusive, depending on the theoretical and methodological basis of the TP as well as the contextual or behavioral factors involved (Arnocky et al., 2014; Bruderer Enzler, 2015; Kooij et al., 2018; Mohammed and Marhefka, 2020).

### Willingness to pay for pro-environmental products and services

Over the past decades, researchers have used a variety of terms to describe pro-environmental behavioral tendencies, from a very specific object of behavior, for example showering time or using biofuels, to more abstract and broad multi-dimensional constructs, such as environmental concern (Lange and Dewitt, 2019). This study uses willingness to pay for pro-environmental products and services as the behavioral context or outcome. Previous research has found that consumers with more favorable environmental values, attitudes, or intentions are more willing to pay for sustainable products and services (Zhang et al., 2020; Qian et al., 2021). Whether and how the different time perspective constructs are related to individuals’ willingness to pay has not been sufficiently documented in the environmental psychology or consumer behavior literature (one exception is Tan et al., 2019). Joireman and Liu (2014) found a positive correlation (0.34) between the CFC-future time perspective and the willingness to pay higher prices for products and services to reduce global warming, while the correlation with the present time perspective (CFC-immediate) was negative (−0.19). We expect that individuals with a future time focus/attention and importance will be willing to pay more for environmentally friendly products, whereas those focused on living in the present will not be willing to pay (much) more for similar goods and services.

The most frequently used theoretical approach for explaining social dilemmas in pro-environmental behavior is based on personal value theories (Stern, 2000; Milfont and Gouveia, 2006; de Groot and Steg, 2008; Joireman and King, 2016). This study will use two convergent or conflicting (dual) dimensions of personal value (hedonic and utilitarian) to find possible structural relationships between constructs to compare the validity of time focus and time importance when measuring future time perspective.

### Personal values, social dilemmas, and pro-environmental behavior

In contrast to beliefs and attitudes, core values as guiding principles in life are broad and stable and transcend specific actions and situations (Schwartz, 2012). Personality traits, defined as tendencies to show consistent patterns of thoughts, feelings, and behavioral tendencies, are different from core values, which are considered to be more motivational in their origin (Parks-Leduc et al., 2015). Traits with a biological basis can influence values, while values are also influenced by other internal and external sources, including norms and culture (Parks-Leduc et al., 2015). These thoughts have led to a debate on whether one’s time perspective is a stable, general, and independent personality trait (Kairys and Liniauskaite, 2015) or a general motivational value-like construct (Milfont and Gouveia, 2006; Joireman and Liu, 2014), including a dynamic, situation-dependent, and flexible concept of motivation that reflects attitudes, cognition, planning, and goals (Shipp et al., 2009; Worrell et al., 2013; Kooij et al., 2018). We discuss the two theories from a social dilemma perspective.

For decades, the social dilemma, which is essentially a conflict between individual and collective (or social) interests, has been applied as a theoretical framework in studies of economic, sustainable, and environmental behavior (see Olsen and Tuu, 2021 for a recent update). For example, Khachatryan et al. (2013) established a consumer preference for biofuels within the context of a three-dimensional social dilemma framework recognizing a social conflict (individual versus collective), a temporal conflict (immediate versus future interests), and a biospheric conflict (human versus biospheric interests). Research has generally shown that *self-enhancement* values are negatively related whereas *self-transcendence* values are positively related to pro-environmental beliefs, attitudes, and behavior (Stern, 2000). However, the results concerning the strength and valence of the relationships across behavioral contexts are not consistent. The general tendency is that altruistic and biospheric values are positively associated whereas hedonistic and egoistic goals are negatively associated with pro-environmental/sustainable attitudes, intentions, and behavior (Olsen and Tuu, 2021).

Studies have proved that biospheric and hedonistic values are the most robust conflicting values (social dilemma) to explain or predict environmental attitudes, intention, or behavioral tendencies (Steg et al., 2015; Balunde et al., 2019; Thelken and de Jong, 2020). For relevance and simplicity, this study therefore includes biospheric and hedonistic values as the most salient conflicting environmental value dimensions to test and compare the nomological validity of the time focus and the time importance approach. Next, we elaborate on two theoretical approaches.

### Environmental values as antecedents of the future time perspective

The “values—belief/cognition/attitude—intention/behavior” framework (VAB: Stern et al., 1995) is well documented in the environmental and sustainability literature (Steg and Vlek, 2009; Milfont et al., 2010; Milfont and Schultz, 2018). Defining the TP as a cognitive construct, one can expect that core or environmental values influence behavioral tendencies through cognitive or attitude-oriented constructs (Fishbein and Ajzen, 2010). This theoretical approach, along with the theory of planned behavior (Ajzen, 1991), dominates several models designed to explain sustainable consumption and pro-environmental behavior (e.g., de Groot and Steg, 2008; Zhang et al., 2020), including willingness-to-pay (Shin et al., 2017). In addition, Qian et al. (2021) suggested that egoistic and biospheric values are directly related to environmental attitude strength.

Empirical studies have shown that if one defines and measures the CFC perspective of a specific area of behavior (e.g., health behavior or eating behavior), the construct moves closer to the concrete behavior in a nomological hierarchy from personality *via* values toward specific beliefs, attitudes, intentions, and behavior (Murphy et al., 2020; Pozolotina and Olsen, 2020). Other recent studies have suggested that the CFC time perspective can be domain-specific because individuals can be time-oriented in some aspects of life but not in others (McKay et al., 2018; Murphy et al., 2020). Some attitude scholars have suggested that broad bandwidth attitudes and values can predict a wide range of behaviors with relatively low fidelity and accuracy (Ajzen, 2012). Low nomological and predictive validity of this kind is a logical consequence of the bandwidth–fidelity trade-off: broad dispositions are expected to have low fidelity to specific behaviors but a strong relationship with broad categories of behavioral outcomes. In contrast, a narrowly defined construct has the advantage of high fidelity and can predict a closely matched description of behavior more precisely (e.g., Soto and John, 2017). According to Ajzen (2012), this logic can be extended to the relationship between attitudes and values: “Because the bandwidth of broad values is even greater than that of general attitudes, we would expect only modest correlations between values and attitudes” (p. 1). Empirical support for the compatibility principle is solid and consistent (Kraus, 1995; Fishbein and Ajzen, 2010).

Based on the assumption that the TP can be based on beliefs, attitudes, cognitions, and motivations (e.g., the VAB model in the pro-environmental context), we investigate whether time focus (TF) and time importance (TI) mediate the relationship between hedonic/biospheric values and individuals’ willingness to pay for environment friendly products. Individuals who adhere to biospheric values (a facet of self-transcendence) may pay attention to and focus on the benefits for future generations and the environment (e.g., reducing global warming and preventing climate change, saving the earth, etc.) as a trade-off to focusing on the present cost of money, pleasure, or time sacrifice. On the other hand, those with hedonistic values will focus on their present feelings and excitement in the situation and pay less attention to the future. For the terminology to be consistent with the value activation approach (and contrasting the mediation awareness model discussed below), we call this alternative theoretical approach the “Time Perspective Activation Model” (TPAM).

However, hedonic, gain and normative expectations are conflicting (Steg et al., 2014). Individuals behave in an environmentally friendly manner in order to improve one’s self-image or reputation, or feel a warm emotional glow from giving as a reward (Hartmann et al., 2017). Therefore, we anticipate that hedonistic individuals will focus on both the present and the future (e.g., Thelken and de Jong, 2020) but that the hedonic–present relationship will be stronger than the hedonic–future relationship. Unlike Olsen and Tuu (2021), we define the TP as a general construct with no specific association with pro-environmental behavior or energy use.

### Future time perspective as an antecedent of environmental values

Using the ZTPI framework, a few studies have found a relationship between values and the TP (Milfont and Gouveia, 2006). Khachatryan et al. (2013) claimed to be the first to show a relationship between egoistic, altruistic, and biospheric values and CFC (present and future). Joireman and Liu (2014) used the Value–Belief–Norm (VBN) model of pro-environmental behavior (Stern, 2000) and proposed a *mediation-awareness model* assumes that CFC leads individuals to be aware of the long-and short-term consequences of their actions and to anticipate those consequences, and this awareness influencing their behavior. Thus, by defining the TP as a broad and stable dispositional trait-like construct (Kairys and Liniauskaite, 2015) with no associations with the broad-bandwidth perspective (Ajzen, 2012), it is possible to adopt Joireman and Liu (2014) mediation-awareness model, assuming that CFC leads individuals to be aware of the long-and short-term consequences of their actions. We call this theoretical approach the “Value Activation Model” (VAM) and use it to determine if the TP can activate the core environmental values. Theoretically, this approach is similar to the Norm Activation Model (NAM: de Groot and Steg, 2009). We suggest that activation encompasses awareness, focus, importance, and other aspects of engagement and activation.

Bruderer Enzler et al. (2019) investigated the relationships between environmental concern (a composite construct of concern, attitudes, opinions, and actions), CFC, and household electricity use. They explored whether the effect of future orientation on electricity use could be mediated by environmental concern, which corresponds to the “awareness model” mentioned earlier (Joireman et al., 2006). Their results did not support the mediation effect of CFC on electricity use behavior. Other studies have found a link between environmental values (egoistic/hedonic and biospheric), CFC, and environmental attitudes/behavior without modelling the associations between values and the TP (Doran et al., 2017; Thelken and de Jong, 2020). It is worth noting that all the previous studies on values–TP relationships have used either the ZTPI or the CFC approach (or both) and defined and measured the TP as general, broad, and stable patterns of individual behavioral traits and emotional, attitudinal, and other psychological phenomena (Kairys and Liniauskaite, 2015).

**Figure 1:**
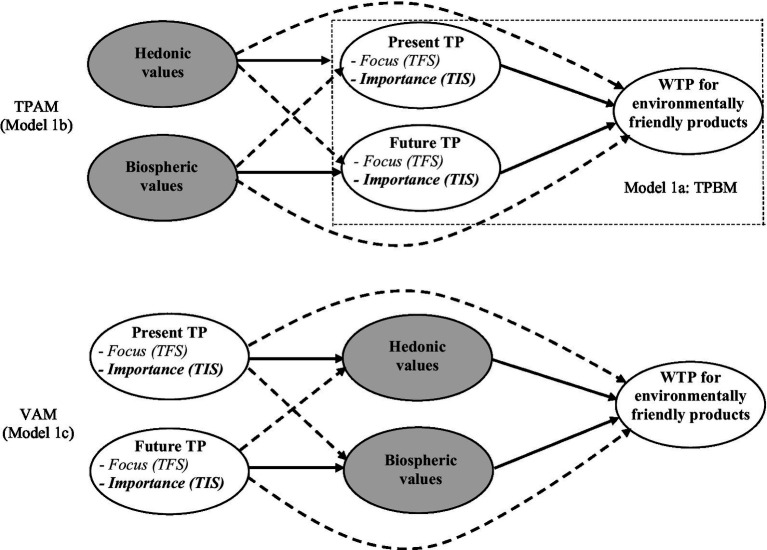
Alternative models for comparing time focus and time importance. Model 1a: Time perspective behavorial model (TPBM in the rectangle). Model 1b: Time perspective activation model (TPAM—total figure). Model 1c: Value activation model (VAM).

In summary, this study uses two different cognitive scales to assess future time perspectives: the Temporal Focus Scale (TFS: Shipp et al., 2009) and the new Time Importance Scale (TIS) that is based on the importance dimension of attitude strength theory (Howe and Krosnick, 2017). The Time Perspective Behavioral Model (TPBM) is shown as Model 1a in the rectangle in Figure 1. It only tests the direct relationship between the future time perspective and the willingness to pay for environmentally friendly products. It advances some simple correlational studies by using structural equation modelling and thus controls for measurement errors. This model allows us to compare the relationships with those reported in previous correlational studies about the predictive validity of other TP scales (e.g., CFC and ZTPI) concerning pro-environmental outcomes. Many researchers have advocated testing models with alternative structures when examining predictive and nomological validity in cross-sectional survey (Bagozzi and Yi, 1988; Rindfleisch et al., 2008). This study proposes and compares two competing nomological theoretical models to explore the discrepancies in the theoretical discussion about definitions, operationalization, and possible relationships between environmental values and time perspectives. The first competing model (Extended 1b, Figure 1) presents a theory whereby values activate time perspectives (TFS and TIS) and serve as a mediator between environmental values and willingness to pay (Time Perspective Activation Model (TPAM)). The second competing model (Model 1c, Figure 1) proposes an alternative approach whereby the TP activates values (Value Activation Model (VAM)).

## Methods

### Participants and construct measurement

In June 2020, a worldwide online survey research company (YouGov Norway AS) administered a questionnaire to a nationwide representative sample of Norwegian consumers, consisting of 633 adult volunteers ranging in age from 18 to 60+ years, of whom 52% were male and 48% were female. The sample was proportionally distributed by location, education, and status, with 58% of the respondents having a college degree at a minimum. There was no formal pre-registration for this survey. However, the theory, formulation of hypotheses, operational definitions, scales of the variables to be used, and a detailed description of how the sample was to be drawn and composed were established before actual data analysis. Our sample size fulfills conservative rule-of-thumb requirements for structural equation models (e.g., *n* > 500, DeVellis and Thorpe, 2022; 10 observations per indicator variable; Wang and Wang, 2012). Our sample size also satisfies the studies based on Monte Carlo simulation that generally recommend *n* = 200 (Boomsma and Hoogland, 2001) as a minimum sample size for SEM research. In addition, based on Preacher and Coffman (2006) model-based sample, we calculated the required sample size for an RMSEA = 0.05, a statistical power level of 80%, and a probability level of 5% to be 103 observations. Our sample is well above this limit, fulfilling the conditions required.

The measuring instruments were adapted from previous research and modified to fit the needs of the current study. The questionnaire was translated from English into Norwegian. Most items in the questionnaire were arranged in random order to eliminate response order effects. We used the *Temporal Focus Scale* (TFS: Shipp et al., 2009) to assess the cognitive engagement with or attention to the present (e.g., I live my life in the present) and the future (e.g., I focus on my future), respectively, with a four-item validated scale. The TFS items were rated on a 7-point scale, where 1 = never, 3 = sometimes, 5 = frequently, and 7 = constantly. The respondents were asked to think about their present and future in general before answering each question in the order presented by Shipp et al. (2009). This scale has been validated in several studies across cultures and age groups (e.g., Chishima et al., 2017: PID). According to Mohammed and Marhefka (2020), the TFS has the strongest validation properties among the scales that are most often used to assess TPs in the area of organizational behavior (i.e., the TFS, ZTPI, CFC, and Balanced Time Perspective).

*Attitude importance* reflects the degree or priority that a person attaches to an attitude object and is most frequently measured by asking respondents to report how significant the attitude object is to them, how concerned they are about it, or how much they care, think, or gather information about it (Howe and Krosnick, 2017). The two most frequently used items to assess attitude importance are (1) “How important is this issue for you personally?,” anchored from not at all important to extremely important, and (2) “How much do you personally care about this issue?,” anchored from not at all to very much (e.g., Wegener et al., 1995; Visser et al., 2003). Our study used these two items, in addition to “relevance” (irrelevant to very relevant to me), measured on a 7-point rating scale. The last item has frequently been used to assess the importance of, commitment to, involvement with, and engagement with an attitude object in the consumer psychology literature (e.g., Zaichkowsky, 1994). The object/issue of the evaluation was the “present” Balanced Time Perspective—framed as “Living for the moment/present is ..”—and the “future”—framed as “Care about the future consequences is ...” Thus, our study did not use the conventional method of developing a scale for measurement and validation of psychological constructs (DeVellis and Thorpe, 2022), because attitude importance is based on a well-defined theoretical construct in attitude strength theory (Howe and Krosnick, 2017). The different time frames (present vs. future) are defined as different attitude objects (Eagly and Chaiken, 1993) and operationalized with “generic” items for assessing (personal) importance (Boninger et al., 1995, p. 160).

Following Schwartz (2012), *values* were assessed by asking the respondents to rate the importance of their values as guiding principles in their lives on a 9-point scale ranging from opposed to my principles to extremely important. *Hedonic values* (HVs) were measured with three items (pleasure, enjoying life, and gratification for oneself) and *biospheric values* (BVs) with four items (protecting the environment, respecting the earth, uniting with nature, and preventing pollution). Those items have previously been validated for assessment of HVs and BVs by several scholars in environmental behavior (e.g., Steg et al., 2014; Balunde et al., 2019; Thelken and de Jong, 2020).

In the literature, there are both direct and indirect approaches to assessing consumers’ *willingness to pay* (WTP) for products (Miller et al., 2011). In practice and in consumer research, most marketing researchers favor the *direct* approach, asking respondents in surveys to evaluate their WTP for products and services in closed-or open-ended question formats. The WTP for environmentally friendly products was assessed by Tully and Winer (2014) and Shin et al. (2017) as the desire to pay an extra percentage over the regular price, and this method tends to be reasonably robust (Schmidt and Bijmolt, 2020). We implemented this scale by asking the respondents the following question: “How much more are you willing to pay for products and services that are environmentally friendly?” We offered them nine response options (< 5%, 5–10%, 11–15%, 16–20%, up to more than 40%) for each of the following three product and service categories: energy (electricity, oil, and gas), clothing, and airline services. Energy, transport, and clothing/fashion are considered to be three of the four most polluting industries in the world. In this study, the combination of the three main categories of goods and services indicates environmentally friendly behavior/outcomes. These items have previously been used in scholarly articles to indicate attitude objects concerning environmental energy conservation, transition, or saving behavior as well as climate change (e.g., Steg et al., 2015; Zhang et al., 2020). See Carrus et al. (2021) for a recent review. The scales of measurement and their properties are presented in Table 1.

**Table 1:** Confirmatory factor analysis.

Constructs and items	Factor loadings	*t*-values	CR	AVE
*Temporal focus: present (TFP)*				
I focus on what is currently happening in my life	0.70^***^	18.77	0.83	0.55
My thoughts are here and now	0.80^***^	22.41		
I think about where I am today	0.78^***^	21.56		
I live my life in the present	0.67^***^	17.79		
*Temporal focus: future (TFF)*				
I think about what my future holds	0.90^***^	28.03	0.91	0.71
I think of times to come	0.90^***^	28.27		
I focus on my future	0.83^***^	24.72		
I focus on what tomorrow will give me	0.70^***^	19.44		
*Temporal importance: living in the present (TIP)*				
Not at all important—very important	0.88^***^	26.97	0.88	0.71
Means nothing—means a lot	0.85^***^	25.30		
Irrelevant—very relevant to me	0.80^***^	23.31		
*Temporal importance: considering future consequences (TIF)*				
Not very important—very important	0.90^***^	28.59	0.92	0.80
Means nothing—means a lot	0.88^***^	27.41		
Irrelevant—very relevant to me	0.91^***^	28.87		
*Hedonic values (HVs)*				
Pleasure	0.83^***^	23.51	0.83	0.61
Enjoying life	0.80^***^	22.42		
Gratification for oneself	0.71^***^	19.14		
*Biospheric values (BVs)*				
Pollution prevention: protecting natural resources	0.87^***^	27.11	0.91	0.71
Respecting the earth: harmony with other species	0.84^***^	25.55		
Unity with nature: to fit into nature	0.78^***^	22.56		
Protecting the environment: conserving nature	0.87^***^	27.06		
*Willingness to pay (WTP)*				
Energy (electricity, fuel, etc)	0.86^***^	26.19	0.91	0.76
Airline travel	0.87^***^	26.54		
Clothing	0.89^***^	27.67		

*** *p*-value < 0.001; model fit: chi-square = 594.85, df = 230, *p*-value < 0.001; GFI = 0.926; CFI = 0.964; RMSEA = 0.050.

### Analytical procedures

We used structural equation modelling with latent variables in AMOS 24.0 (Arbuckle, 2015) to perform the analyses. We ran a confirmatory factor analysis (CFA) to examine the significance and convergent validity of the items and the discriminant validity of the constructs. Next, we tested three competing models, depicted in Figure 1, with two alternative TP scales: the TFS and the TIS. Based on Edwards & Lambert, 2007 general path analysis framework, we tested the mediator effect of hedonic and biospheric values in the path of T value of ps-WTP relationships (TPAM, Figure 1 Model 1b) and the mediator effect of present and future focus/importance in the path of values-TP-WTP relationships (VAM, Figure 1 Model 1c), respectively. We applied chi-square difference tests comparing a constrained versus an unconstrained model to examine the strength of the relationship between hedonic and biospheric values and time perspective constructs (i.e., a test of the strength/magnitude of asymmetric effects). At each stage, the model fit was evaluated using three standard metrics: the goodness-of-fit index (GFI), the comparative fit index (CFI), and the root mean square error of approximation (RMSEA). Good models require the *GFI* and *CFI* indices to be above 0.90 and the RMSEA to be lower than 0.08 (Hair et al., 2014).

## Results

### Confirmatory factor analysis and convergent and discriminant validity

The measurement models as well as the measurement scales and the model goodness-of-fit statistics are shown in Table 1. All factor loadings are high and significant (*value of p*s <0.001), and the composite reliability (*CR*) and average variance extracted indices (*AVE*) are superior to 0.70 and 0.50, respectively, providing evidence of convergent validity (Hair et al., 2014). The *AVE* value for each construct is greater than the bivariate squared correlations with other constructs, reflecting discriminant validity (Fornell and Larcker, 1981; Table 2).

**Table 2:** Means, standardized deviations, and intercorrelations.

	Mean	SD	TFP	TFF	TIP	TIF	HV	BV	WTP
Temporal focus: present (TFP)	4.46	0.98	*0*.*55**^a^*	0.30	0.50	0.22	0.43	0.25	0.06^ns^
Temporal focus: future (TFF)	4.27	1.16	0.32	*0*.*71**^a^*	0.10	0.40	0.23	0.30	0.27
Temporal importance: present (TIP)	5.47	1.18	0.51	0.14	*0*.*71 ^a^*	0.49	0.58	0.31	-0.03^ns^
Temporal importance: future (TIF)	5.53	1.28	0.24	0.43	0.51	*0*.*80 ^a^*	0.38	0.56	0.19
Hedonic values (HVs)	7.01	1.37	0.44	0.31	0.60	0.43	*0*.*61 ^a^*	0.41	-0.02^ns^
Biospheric values (BVs)	6.59	1.61	0.27	0.33	0.32	0.57	0.45	*0*.*71 ^a^*	0.33
Willingness to pay (WTP)	5.48	2.06	0.04^ns^	0.16	-0.07^ns^	0.13	−0.11	0.26	*0*.*76 ^a^*

a The AVE for each construct is reproduced on the main diagonal; the intercorrelations among the latent constructs are shown below the main diagonal; and the intercorrelations among latent constructs for testing the common method variance model are shown above the main diagonal.

^ns^*p* > 0.05. The bivariate correlations are significant at minimum 5% level.

### Testing for common method variance

Because all the data were self-reported and collected through a single questionnaire, we tested for the presence of the common method effect, which is an extension of discriminant validity. Method effects represent the bias that can result from using the same method to assess different traits (Podsakoff et al., 2003). We evaluated this bias by estimating a measurement model with a single-method first-order factor added to the basic measurement model (Podsakoff et al., 2003). The fit of the CFA model with the common method factor was better (*χ^2^* = 488.73, *df* = 208, *p* < 0 0.001; *GFI* = 0.940; *CFI* = 0.972; *RMSEA* = 0.046) than that of the basic measurement CFA model (*χ^2^* = 594.85, *df* = 230, *p* < 0 0.001; *GFI* = 0.926; *CFI* = 0.964; *RMSEA* = 0.050). However, the factor loadings within each construct and the intercorrelations between the constructs remained nearly unchanged (Table 2). The results indicate that the presence of the systematic measurement error is not of concern.

### Direct relationships between time perspective and willingness to pay

The first model, the Time Perspective Behavioral Model (TPBM: Model 1a in Figure 1), focuses solely on the link between the TP and the WTP after controlling for demographic characteristics. We used this model to compare our results with previous correlational studies on the bivariate relationship between time perspective scales (e.g., CFC and ZTPI) and pro-environmental outcomes. The goodness-of-fit statistics for both the TIS (*GFI* = 0.956; *CFI* = 0.967; *RMSEA* = 0.055; *R^2^* = 8.5%) and the TFS (*GFI* = 0.942; *CFI* = 0.950; *RMSEA* = 0.059; *R^2^* = 7.4%) are satisfactory, and the explained variance in the WTP is slightly in favor of the TIS. The coefficients presented in Table 3 show that the TIS’s present and future dimensions relate significantly to the willingness to pay for pro-environmental products and services (*β* = −0.16 and *β* = 0.18, respectively). In turn, only the TFS’s future dimension relates significantly to the willingness to pay (*β* = 0.12). The effects of demographic characteristics, such as age (*β* = −0.16) and education (*β* = 0.14) are significant, whereas gender, marital status, and weight do not relate significantly to the willingness to pay.

**Table 3:** Comparing the predictive validity of the TFS and the TIS for willingness to pay (WTP) in the time perspective behavioral model (TPBM).

Constructs	Temporal Focus Scale (TFS)		Temporal Importance Scale (TIS)	
	Std Beta	*t*-values	Std Beta	*t*-values
Present → willingness to pay	0.02	0.33^ns^	−0.16	−3.16**
Future → willingness to pay	0.12	2.07**	0.18	3.63***
*Controlled variables*				
Age	−0.18	−4.19***	−0.18	−4.16***
Gender	−0.08	−1.81^ns^	−0.07	−1.69^ns^
Married status	0.03	0.67^ns^	0.04	0.87^ns^
Education	0.14	3.55***	0.14	3.41***
Weight	−0.03	−0.68^ns^	−0.03	−0.67^ns^
*Fit indices*				
Chi-squared (df)	309.04 (97)		206.64 (71)	
*GFI*	0.942		0.956	
*CFI*	0.950		0.967	
*RMSEA*	0.059		0.055	
*R^2^* (willingness to pay)	7.4%		8.5%	

ns *p* > 0.05.

*p < 0.05; **p < 0.01; ***p < 0.001.

### Comparing time perspective activation model (TPAM) and value activation model (VAB)

**Figure 2:**
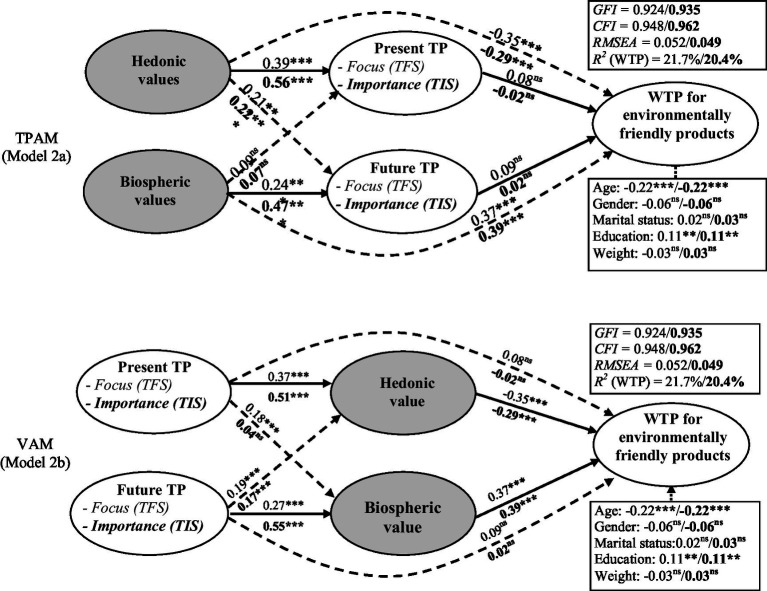
Results from alternative models for comparing time focus and time importance. Model 2a: The time perspective activation model (TPAM). Value for the Temporal Importance Scale (TIS) are in bold. ns, non-significant, *p < 0.05, **p < 0.01, ***p < 0.001. Model 2b: The estimated model of the value activation model (VAM). ns, non-significant, *p < 0.05, **p < 0.01, ***p < 0.001.

Model 1b (TPAM) and Model 1c (VAB) integrate values as mediators and, respectively, antecedents of the relationship tested in Model 1a (TPBM), respectively. Figure 2 and Table 4 summarize the main results of the structural relationships and control variables in these models, and Supplementary Appendix A presents a test of the relative strength of the (asymmetric) relationships in the two different models. A comparison of the models using the two scales revealed that the goodness-of fit statistics are satisfactory for both scales: TIS (*GFI* = 0.935; *CFI* = 0.962; *RMSEA* = 0.049; *R^2^* = 20.4%) and TFS (*GFI* = 0.924; *CFI* = 0.948; *RMSEA* = 0.052; *R^2^* = 21.7%).

**Table 4:** Testing of structural relationships and control variables in the TPAM and VAM.

Time perspective activation model (TPAM)			Value activation model (VAM)		
Paths	Standardized coefficients	*t*-values	Paths	Standardized coefficients	*t*-values
*Direct effects*					
HV → future	0.21	4.14***	Future → HVs	0.19	4.24***
	0.22	4.95***		**0.17**	3.65***
HV → present	0.39	7.17***	Present → HVs	0.37	7.41***
	0.56	11.28***		**0.51**	10.17***
BV → future	0.24	5.04***	Future → BVs	0.27	6.12***
	**0.47**	10.89***		**0.55**	11.76***
BV → present	0.09	1.92^ns^	Present → BVs	0.18	3.88***
	0.07	**1.63**^ns^		**0.04**	**0.95**^ns^
HV → WTP	−0.35	−6.36***		−0.35	−6.36***
	**−0.29**	−4.76***		**−0.29**	−4.76***
BV → WTP	0.37	7.49***		0.37	7.49***
	0.39	7.00***		**0.39**	7.00***
Future → WTP	0.09	1.92^ns^		0.09	1.92^ns^
	0.02	**0.34**^ns^		**0.02**	**0.34**^ns^
Present → WTP	0.08	1.61^ns^		0.08	1.61^ns^
	−0.02	**−0.04**^ns^		**−0.02**	**−0.41**^ns^
***Indirect effects***					
HVs → future → WTP	0.021/**0.002** (0.011/**0.012**)^a^	1.91^ns^	Future → HVs → WTP	−0.066/**−0.049** (0.018/**0.020**)^a^	−3.69***
		**0.17**^ns^			2.45**
HVs → present→ WTP	0.039/**−0.010** (0.022/**0.034**)^a^	1.77^ns^	Present → HVs → WTP	−0.129/**−0.148** (0.028/**0.033**)^a^	4.63***
		**−0.29**^ns^			4.48***
BVs → future → WTP	0.022/**0.005**	1.83^ns^	Future → BVs → WTP	0.099/**0.215** (0.017/**0.036**)^a^	5.88***
	(0.012/**0.025**)^a^	**0.20**^ns^			5.96***
BVs → present → WTP	0.009/**−0.001** (0.007/**0.005**)^a^	1.29^ns^	Present → BVs → WTP	0.067/**0.017** (0.018/**0.018**)^a^	3.70***
		**−0.20**^ns^			**0.87**^ns^
***Controlled variables***					
Age → WTP	−0.22	−5.41***		−0.22	−5.41***
	**−0.22**	−5.40***		**−0.22**	−5.39***
Gender → WTP	−0.06	−1.53^ns^		−0.06	−1.53^ns^
	**−0.06**	**−1.49**^ns^		**−0.06**	**−1.49**^ns^
Marital status → WTP	0.02	0.63^ns^		0.02	0.63^ns^
	0.03	**0.73**^ns^		**0.03**	**0.74**^ns^
Education → WTP	0.11	2.85**		0.11	2.85**
	0.11	2.89**		**0.11**	2.88**
Weight → WTP	−0.03	−0.66^ns^		−0.03	−0.66^ns^
	**−0.03**	**−0.65**^ns^		**−0.03**	**−0.63**^ns^
***Fit indices***					
Chi-squared (*df*)	579.82 (216)/**446.82 (176)**			579.82 (216)/**446.82 (176)**	
GFI	0.924/**0.035**			0.924/**0.935**	
CFI	0.948/**0.962**			0.948/**0.962**	
RMSEA	0.052/**0.049**			0.052/**0.049**	
AIC	699.823/**556.818**			699.823/**556.812**	
R^2^ (WTP)	21.7%/**20.4%**			21.7%/**20.4%**	

a The standardized errors of indirect effects were estimated using bootstrapping.

HVs, hedonic values; BVs, biospheric values; WTP, willingness to pay. The results for the Temporal Importance Scale are in **bold**. ^ns^*p* > 0.05; *p < 0.05; **p < 0.01; ***p < 0.001.

For the TPAM model (Model 1b), biospheric values have significant relationships with future focus and importance (*β* = 0.24, *t* = 5.04, *p* < 0.001 and *β* = 0.47, *t* = 10.89, *p* < 0.001, respectively), but non-significant associations with present focus and importance (*β* = 0.09, *t* = 1.92, *p* > 0.05 and *β* = 0.07, *t* = 1.63, *p* > 0.05, respectively). The hedonic values have significant relationships with future focus and importance (*β* = 0.21, *t* = 4.14, *p* < 0.001 and *β* = 0.22, *t* = 4.95, *p* < 0.001, respectively) as well as with present focus and importance (*β* = 0.39, *t* = 7.17, *p* < 0.001 and *β* = 0.56, *t* = 11.28, *p* < 0.001, respectively). However, the effects of future focus (*β* = 0.09, *t* = 1.92, *p* > 0.05) and importance (*β* = 0.02, *t* = 0.34, *p* > 0.05) on WTP are not significant. The same findings apply to present focus (*β* = 0.08. *t* = 1.61, p > 0.05) and importance (*β* = −0.02, *t* = −0.04, *p* > 0.05).

Hence, we found that all expected indirect effects are statistically insignificant in the TPAM model, regardless of the scale employed (TIS or TFS). We also noticed that the TIS and the TFS scales have distinct relationships in the nomological network. The comparative pairs of the associations between values and times constructs, as shown in Supplementary Appendix B, reveal significant differences for all TIS instances but just half of the TFS cases. The difference in the association between hedonic values and future importance vs. present importance, for example, is significant (*β* difference = 0.34; *χ^2^* difference *=* 37.29, *p* < 0.001, *df = 1*) while the counterpart difference for future focus vs. present focus is non-significant (*β* difference = 0.18; *χ^2^* difference *=* 1.66, *p* > 0.05, *df = 1*).

For the VAM (Model 1c), both future focus and importance are significantly associated with biospheric values (*β* = 0.27, *t* = 6.12, *p* < 0.001 and *β* = 0.55, *t* = 11.76, *p* < 0.001, respectively) and hedonic values (*β* = 0.19, *t* = 4.24, *p* > 0.05 and *β* = 0.17, *t* = 3.65, *p* < 0.001, respectively). In addition, both present focus and importance are significantly associated with hedonic values (*β* = 0.37, *t* = 7.41, *p* < 0.001 and *β* = 0.51, *t* = 10.17, *p* < 0.001, respectively). Present focus is significantly associated with biospheric values (*β* = 0.18, *t* = 3.88, *p* < 0.001), but present importance is not (*β* = 0.04, *t* = 0.95, *p* > 0.05). Importantly, the effects of hedonic values on WTP are significant for both the TFS and the TIS (*β* = −0.35, *t* = −6.36, *p* < 0.001 and *β* = −0.29, *t* = −4.76, *p* < 0.001, respectively). Similarly, the relationship between biospheric values and WTP is significant for both the TFS (*β* = 0.37, *t* = 7.49, *p* < 0.001) and the TIS (*β* = 0.39, *t* = 7.00, *p* < 0.001). Except for a non-significant indirect relationship of present importance with WTP *via* biospheric values (present importance—biospheric values—WTP: *β* = 0.017, *t* = 0.87, *p* > 0.05), all the indirect relationships are significant in the VAM models.

The TFS and TIS share similar roles within the proposed VAM models. However, the TIS scale, in most cases, shows better nomological validity than the TFS scale in the associations with environmental values and in a social dilemma direction (perspective), as illustrated in Supplementary Appendix B. For example, the difference in the strength of the associations between future importance and biospheric vs. hedonic values (*β* difference = 0.38; *χ^2^* difference *=* 66.99, *p* < 0.001, *df = 1*) is significantly larger than the counterpart difference for future focus (*β* difference = 0.08; *χ^2^* difference *=* 7.81, *p* < 0.001, *df = 1*).

## Discussion and conclusion

The theoretical foundation of this research is the attitude strength literature (Eaton and Visser, 2008; Howe and Krosnick, 2017). We defined *time importance* as a subjective sense of the concern, caring, and significance that the individual attaches to different periods. *Time importance* is an alternative to other cognition-based definitions of time perspectives, such as the *time focus* suggested by Shipp et al. (2009) and the *attitude time–frequency/time orientation* proposed by Worrell et al. (2013). Those measurement approaches share the same theoretical perspectives, suggesting that individuals can allocate their cognitive attention and attitudes to various time targets: the priority and concern that individuals attach to the different time attitudes. As part of the attitudes, *time importance* has a positive and negative valence and differs in extremity and strength. This study proposed to validate the nomological validity of the *Time Importance Scale* (TIS) and to compare it with *the Temporal Focus Scale* (TFS: Shipp et al., 2009). The two scales are based on the same theoretical cognitive foundations, are short and easy to manage, and are theoretically more focused than some broadly defined scales, such as the CFC and ZTPI (Mohammed and Marhefka, 2020). This study extends the application of the TFS into the area of environmental behavior and its relationship to environmental values from a social/time dilemma perspective.

Individuals’ evaluation of (time) importance influences their thoughts, emotions, intentions, and behavior (Eaton and Visser, 2008; Howe and Krosnick, 2017). As a cognitive and knowledge-based attitude construct and scale, time importance depends on specific situations, actions, and context (Fishbein and Ajzen, 2010; Ajzen, 2012). Thus, the definition and corresponding assessment can be flexible because individuals can allocate their time focus, attention, and attitude importance to different contextual levels: as time in general, as the future time perspective in general, or as the (future) time perspective associated with different behavioral outcomes, such as sustainable behavior (as in this study), health and eating behavior, or financial and/or organizational behavior, to name a few examples. This study defined time importance (TIS) at the general time level, analogous to the general time focus (TFS). However, WTP was defined and measured as a specific indicator of (environmental) behavior. Thus, following the theoretical argumentations within the principle of compatibility and broad bandwidth attitude or behavior (Ajzen, 2012; Soto and John, 2017), this study used the “worst-case” approach to test for predictive validity between the TP and the environmental outcome (Lange and Dewitt, 2019).

This study performed confirmatory factor analyses to validate the theoretical constructs. The results confirmed the convergent and discriminant validity. The correlation between the TFS and the TIS present is 0.51 (*p* < 0.001) and the correlation between the TFS and the TIS future is 0.43 (*p* < 0.001). The correlations between the present and the future time perspective for the TFS and the TIS are 0.32 (*p* < 0.001) and 0.51 (*p* < 0.001), respectively. These results indicate that the associations between the scales correspond to a common core (attention to time) and a separate core (present versus future). Studies of the relationships between the future time perspective (FTP) and pro-environmental behavioral tendencies (Corral-Verdugo et al., 2006; Milfont and Gouveia, 2006; Arnocky et al., 2014; Bruderer Enzler, 2015; Doran et al., 2017; Kooij et al., 2018) have provided mixed results and in several cases low or non-existent predictive validity (McKay et al., 2018). This study confirmed our expectations of a low and significant negative relationship between the present time perspective and willingness to pay for environmentally friendly products and a significant positive relationship between the future time perspective and willingness to pay for environmentally friendly products. These results were confirmed with the TIS and partially confirmed (only for the future) with the TFS when we performed a simple FTP → WTP structural model without including other constructs (antecedents or moderators). When we included the values in the model, however, neither the TIS nor the TFS was significantly related to willingness to pay for environmentally friendly products.

For decades, social dilemmas, and conflicts between individual and collective interests and values, have been a strong theoretical framework for sustainable and environmental behavior (Khachatryan et al., 2013; Olsen and Tuu, 2021). In particular, the conflict between biospheric and hedonic values suggests that collective interests are positively associated with pro-environmental behavioral tendencies, whereas the association of individual interests are almost non-existent or negative (Steg et al., 2014; Balunde et al., 2019; Thelken and de Jong, 2020). Thus, this study validates the nomological validity of the TFS and TIS by proposing two conflicting structural models to test the relationships between pro-environmental values, time perspectives, and pro-environmental outcomes. The first theoretical approach presents the Time Perspective Activation Model (TPAM: values-TP WTP), in which values activate time perspectives (TFS and TIS), which, in turn, serve as direct precursors of the willingness to pay for pro-environmental products. The second model, the Value Activation Model (VAM: TP—value—WTP), proposes an alternative approach in which time perspectives activate values and thus only indirectly influence the willingness to pay for pro-environmental products through activated values.

The results indicate that the VAM fits the data best. This result confirms the previous study by Joireman and Liu (2014) on future versus immediate consequences of the CFC in a context of willingness to pay to reduce global warming as well as one of their earlier studies of the relationship between CFC, social value orientation, and pro-environmental intention and behavior (Joireman et al., 2001). Similarly, the study by Bruderer Enzler et al. (2019) provides mixed results (Bruderer Enzler, 2015) but with environmental concern, rather than values, as the mediating construct. However, in some previous empirical studies (e.g., Milfont and Gouveia, 2006; Arnocky et al., 2014: Doran et al., 2017; Murphy et al., 2020; Olsen and Tuu, 2021), the value–attitude–behavior theory (VAB: Stern et al., 1995) applied to pro-environmental contexts (Steg and Vlek, 2009; Milfont et al., 2010) and attitude strength theories (Boninger et al., 1995; Fishbein and Ajzen, 2010; Howe and Krosnick, 2017) have suggested arguments for the alternative model in which environmental values activate the time perspective, and the time perspective acts as a mediator between values and environmental attitudes, intention, or behavior. Most studies confirming significant relationships between the future time perspective and pro-environmental behavior have used a broad time perspective approach (CFC and ZTPI) or assessed the time perspective linked to the behavioral domain (e.g., Olsen and Tuu, 2021).

Values are beliefs that underlie specific attitudes and form the basis for evaluations (Schwartz, 2012). Traits are consistent patterns of thinking, feeling, and behaving across time and situations (Parks-Leduc et al., 2015). Thus, our findings (VAM: Model 1c/2b) indicate that TP as a general trait can influence values (Joireman and Liu, 2014). Our study defines and measures TP as an association toward time (present/future) in general. At the same time, value dimensions are highly relevant for environmental goals (de Groot and Steg, 2008), and the outcomes (WTP) are measured as a specific indicator of (environmental) behavior. An alternative explanation for the findings indicating that the VAM model fits the data best is the principle of compatibility or that broad bandwidth attitudes are less associated with context-specific constructs or outcomes (Ajzen, 2012; Soto and John, 2017).

This study extends the previous literature by testing the relationship between environmental values, the TFS and the new TIS. Based on other time perspective models such as CFC and ZTPI using the social dilemma principle (Joireman et al., 2001; Milfont and Gouveia, 2006; Khachatryan et al., 2013), the future is associated with biospheric values and the present with hedonic values. Those associations are stronger for time importance than for time focus in our study, implying that the nomological validity of opposing time perspectives and conflicting values is stronger for importance (TIS) than for focus (TFS).

The relationships between the future time perspective and hedonic values are positive and similar for both scales. The findings are supported by others (e.g., Steg et al., 2014; Doran et al., 2017; Thelken and de Jong, 2020) and contradicted by alternative theories of how egoistic or hedonistic individuals can activate pro-environmental behavior (e.g., Hartmann et al., 2017). The relationships between the present time perspective and biospheric values are weak or non-significant for both scales. This result is consistent with prior research (e.g., Olsen and Tuu, 2021), which has found a weak relationship between self-transcendence and CFC-present, supporting the social dilemma (individual versus collective values) and the temporal conflict (present versus future TP) in the context of environmental behavior (Milfont and Gouveia, 2006; Milfont et al., 2012; Khachatryan et al., 2013; Joireman and Liu, 2014). The differences in the theoretical relationships in some cases favor the TIS over the TFS, implying that the new time importance approach is an acceptable alternative to the time focus approach within the cognitive–motivational approach to the future time perspective.

### Limitations and future research

The findings support the Value Activation Model (VAM), in which environmental values play a role as mediators for the effects of time perspectives on environmental outcomes. This indicates that the FTP, when defined and measured as general associations with different time dimensions (present and future), can be treated as a broad and stable dispositional trait-like construct (Zimbardo and Boyd, 1999; Kairys and Liniauskaite, 2015). However, our study used the most salient and contradicted (dual) core environmental values in associations with a specific pro-environmental outcome (WTP for environmentally friendly products). Those domain-specific values outclassed the more general TP in the relationship with WTP. According to some attitude scholars, this relationship can be explained by the bandwidth–fidelity trade-off (Ajzen, 2012) or the compatibility perspective (Fishbein and Ajzen, 2010): broad dispositions of values and attitudes are expected to have low fidelity in relation to specific behavior. Thus, future studies should validate the structural relationships between values, the TP, and environmental outcomes with other value definitions (e.g., self-enhancements versus self-transcendence), domain-specific time perspectives (e.g., the pro-environmental time perspective), and more general/specific framing and approaches to assess environmental outcomes (see Lange and Dewitt, 2019).

In addition, although easy to implement, the scales used in this study include only three items per time dimension. Further research could expand and validate other and more extended versions based on other items of importance (e.g., gathering information) or attitude strength (e.g., ambivalence, accessibility, or certainty; Qian et al., 2021) as well as include the past time perspective in relevant contexts. Future studies could also test those time attitude–strength-related constructs as moderators (Visser et al., 2006) to validate alternative scales of future time perspectives and compare alternative structures between constructs (e.g., values, future–present TP, and moderator effects). Finally, this study used correlation methods based on cross-sectional data; thus, claiming causality is difficult. Experimental or longitudinal designs could be used in future studies to address causality, for instance by testing if priming FTP activates environmental attitudes or if priming environmental attitudes make individuals more concerned about the future.

## Data availability statement

The raw data supporting the conclusions of this article will be made available by the authors, without undue reservation.

## Ethics statement

Ethical review and approval was not required for the study on human participants in accordance with the local legislation and institutional requirements. The patients/participants provided their written informed consent to participate in this study.

## Author contributions

SO put forward the research idea, developed the questionnaire, and prepared the first draft of the theoretical background, review, and discussion part of the manuscript. HT and AT performed the statistical analyses, interpretations of results, and further optimize and complete the manuscript. All authors contributed, read and approved the submitted version.

## Conflict of interest

The authors declare that the research was conducted in the absence of any commercial or financial relationships that could be construed as a potential conflict of interest.

## Publisher’s note

All claims expressed in this article are solely those of the authors and do not necessarily represent those of their affiliated organizations, or those of the publisher, the editors and the reviewers. Any product that may be evaluated in this article, or claim that may be made by its manufacturer, is not guaranteed or endorsed by the publisher.
