# Supplementary material for: Comparing time focus with time importance for measuring future time perspectives in the context of pro-environmental values and outcomes
Source: Front Psychol. 2023 Feb 27;14:945487. doi: 10.3389/fpsyg.2023.945487 (PMC10114413; doi:10.3389/fpsyg.2023.945487)
Supplement: Supplementary file 1 [file Data_Sheet_1.docx]

**APPENDIX A |** Relative strength of relationships in the TPAM and VAM.

| Pairs of compared paths | *Beta*  *difference* | *χ^2^ (df)* | *χ^2^ difference*  *(df = 1) test* |
| --- | --- | --- | --- |
| *The comparative relationships based on the TPAM model* | | | |
| Hedonic values → time focus: present vs future | 0.39 – 0.21  = 0.18 | 581.080 (217)  579.423 (216) | 1.66^ns^ |
| Hedonic values → time importance: present vs future | **0.56 – 0.22**  **= 0.34** | **484.105 (177)**  **446.818 (176)** | **37.29***** |
| Biospheric values → time focus: future vs present | 0.24 – 0.09  0.15 | 590.778 (217)  579.423 (216) | 11.36*** |
| Biospheric values → time importance: future vs present | **0.47 – 0.07**  **= 0.40** | **511.893 (177)**  **446.818 (176)** | **65.08***** |
| Hedonic vs biospheric values → time focus: present | 0.39 – 0.09  0.30 | 597.945 (217)  579.423 (216) | 18.52*** |
| Hedonic vs biospheric values → time importance: present | **0.56 – 0.07**  **= 0.49** | **500.979 (177)**  **446.818 (176)** | **54.16***** |
| Biospheric vs hedonic values → time focus: future | 0.24 – 0.21  = 0.03 | 579.992 (217)  579.423 (216) | 0.57^ns^ |
| Biospheric vs hedonic values → time importance: future | **0.47 – 0.22**  **0.25** | **450.817 (177)**  **446.818 (176)** | **4.00*** |
| *The comparative relationships based on the VAM model* | | | |
| Time focus: present → hedonic vs biospheric values | 0.37 – 0.18  = 0.19 | 583.387 (217)  579.823 (216) | 3.56* |
| Time importance: present → hedonic vs biospheric values | **0.51 – 0.04**  **= 0.47** | **488.059 (177)**  **446.818 (176)** | **41.24***** |
| Time focus: future → biospheric vs hedonic values | 0.27 – 0.19  = 0.08 | 587.631 (217)  579.823 (216) | 7.81** |
| Time importance: future → biospheric vs hedonic values | **0.55 – 0.17**  **= 0.38** | **513.812 (177)**  **446.818 (176)** | **66.99***** |
| Time focus: present vs future → hedonic values | 0.37 – 0.19  = 0.18 | 583.887 (217)  579.823 (216) | 4.06* |
| Time importance: present vs future → hedonic values | **0.51 – 0.17**  **0.34** | **464.711 (177)**  **446.818 (176)** | **17.89***** |
| Time focus: future vs present → biospheric values | 0.27 – 0.18  = 0.09 | 579.831 (217)  579.823 (216) | 0.01^ns^ |
| Time importance: future vs present → biospheric values | **0.55 – 0.04**  **= 0.51** | **484.187 (177)**  **446.818 (176)** | **37.36***** |

*^ns^ p > 0.05; * p < 0.05; ** p < 0.01; *** p < 0.001***; WTP: willingness to pay; values for the Temporal Importance Scale (TIS) are in* ***bold****.*
